# Obesity is associated with an impaired survival in lymphoma patients undergoing autologous stem cell transplantation

**DOI:** 10.1371/journal.pone.0225035

**Published:** 2019-11-08

**Authors:** Sebastian Scheich, Julius C. Enßle, Victoria T. Mücke, Fabian Acker, Lukas Aspacher, Sebastian Wolf, Anne C. Wilke, Sarah Weber, Uta Brunnberg, Hubert Serve, Björn Steffen

**Affiliations:** 1 Department of Hematology and Oncology, University Hospital Frankfurt, Frankfurt am Main, Germany; 2 Department of Gastroenterology, Hepatology, Pulmonology and Endocrinology, University Hospital Frankfurt, Frankfurt am Main, Germany; University of Kentucky, UNITED STATES

## Abstract

Autologous hematopoietic stem cell transplantation (auto-HSCT) provides a potentially curative treatment option for relapsed and refractory lymphomas. Obesity displays an emerging epidemic risk factor for global mortality and is associated with an increased mortality in cancer patients. To date, the impact of obesity on the outcome of lymphoma patients undergoing auto-HSCT is understudied. We conducted a retrospective single-center study assessing 119 lymphoma patients who underwent auto-HSCT. Overall survival (OS) served as the primary endpoint whereas progression free survival (PFS), cumulative incidence of non-relapse related mortality (NRM) and cumulative incidence of relapse were analyzed as secondary endpoints. Obese patients (Body mass index, BMI≥30) had significantly lower OS (45.3% vs. 77.9%; p = 0.005) and PFS (29.8% vs. 67.2%; p<0.001) compared to non-obese patients at 48 months post-transplantation. The cumulative incidence of NRM displayed no significant differences while the cumulative incidence of relapse was significantly increased in patients with BMI≥30 (66.2% vs. 21.5%; p<0.001). Patients with a BMI<25 and overweight patients (BMI 25–30; 76.1% vs. 80.9%; p = 0.585), showed no significant difference in OS, whereas patients with BMI≥30 exhibited significant lower OS when compared to either of both groups (76.1% vs. 45.3%; p = .0.021 and 80.9% vs. 45.3%; p = 0.010). Furthermore, in a multivariate analysis, obesity was identified as an independent risk factor for death (Hazard ratio 2.231; 95% CI 1.024 to 4.860; p = 0.043). Further studies are needed to evaluate the reasons for the higher relapse rate causing higher mortality in obese patients.

## Introduction

Lymphomas represent a heterogenous group of hematological malignancies with diverse phenotypic and molecular features that influence response to therapy and thus outcome. Patients with low- and high-grade lymphomas often experience relapse after initial treatment and require additional therapeutic strategies[1, 2]. In relapsed patients, salvage chemotherapy and subsequent autologous hematopoietic stem cell transplantation (auto-HSCT) with prior high-dose chemotherapy (HDT) provides a potentially curative treatment approach in physiologically fit patients with chemosensitive lymphoma and is the recommended treatment for relapsed high-risk lymphoma patients[3–6].

The incidence of obesity has increased dramatically throughout the last decades. Obesity represents a prominent risk factors in multiple disease conditions as well as for global mortality[7, 8]. Obese patients are at high risk for developing comorbidities such as metabolic syndrome, cardiovascular disease and endocrine disorders[9, 10]. Furthermore, obesity is linked to a general increase in the incidence and mortality of cancer[11–13]. Earlier studies suggest that obesity might also be associated with an increased risk of Non-Hodgkin lymphomas (NHL)[14, 15]. Accordingly, the incidence of the most common lymphoma subtype, diffuse large cell B-cell lymphoma (DLBCL), is increased in patients with higher body mass index (BMI)[16].

Previous studies assessed the outcome of HDT and auto-HSCT in overweight lymphoma patients. However, these studies reached different conclusions concerning survival and onset of relapse[17, 18]. To date, conclusive data concerning the clinical outcome of obese lymphoma patients receiving HDT followed by auto-HSCT is lacking. Therefore, we conducted a retrospective single-center study on 119 lymphoma patients, who were treated with HDT and auto-HSCT to evaluate the impact of obesity on patients’ outcomes.

## Materials and methods

### Study design and definitions

We retrospectively analyzed all consecutive patients in a single center with a lymphoma, who were admitted for auto-HSCT to the Department of Hematology and Oncology of the University Hospital Frankfurt between January 2012 and December 2016 (n = 119). If patients received more than one auto-HSCT, only the first transplantation was included into the analysis.

For the peritransplant period, patients were accommodated on a dedicated transplant unit in air-filtered rooms. A central venous line was routinely inserted on the day of admission. Patients received an anti-infective prophylaxis consisting of fluconazole and levofloxacin until neutrophil engraftment (neutrophil count >500/μl) and cotrimoxazole/trimethoprime and aciclovir were administered until CD4+-cells recovered (>400/μl). In case of fever, blood cultures were collected into appropriate culture bottles (BD BACTEC Lytic/10 Anaerobic/F and BD BACTEC Plus Aerobic/F, Becton Dickinson, Heidelberg, Germany). According to the recommendations of the Center of Disease Control (CDC) a positive culture bottle was defined as blood stream infection (BSI) but if blood cultures were positive for coagulase-negative staphylococci, two consecutive positive cultures were required to define a BSI[19].

Patients received a conditioning regimen based on their lymphoma subtype: carmustine (400 mg/m^2^ day -6) and thiotepa (5 mg/kg twice daily day -5 and day -4) was administered to patients with primary lymphoma of the central nervous system (CNS-lymphoma), carmustine (400 mg/m^2^ day -6), thiotepa (5 mg/kg twice daily day -5 and day -4) and etoposide (150 mg/m^2^ day -5 until day-3) was administered to patients with central and peripheral lymphoma manifestation and all other patients received BEAM (carmustine 400 mg/m^2^ day -7; etoposide 100 mg/m^2^ day-6 until day-3 twice daily, cytarabin 200 mg/m^2^ day-6 until day-3 twice daily and melphalan 140 mg/m^2^ day-2); except one patient suffering from lymphoblastic lymphoma, received thiotepa 4 mg/kg day-6 until day-3 and melphalan 140 mg/m^2^ day-2, one patient with follicular lymphoma received thiotepa 8 mg/kg day -7, etoposide 100 mg/m^2^ day-6 until day-3 twice daily, cytarabin 200 mg/m^2^ day-6 until day-3 twice daily and melphalan 140 mg/m^2^ day-2 [TEAM], and one patient with mantle cell lymphoma received cytarabine 1500 mg/m^2^ d-4 and day-3 twice daily, melphalan 140 mg/m^2^ d-2 and 10 Gray total body irradiation). Neutrophil recovery was supported using (pegylated)-granulocyte-colony stimulating factor.

Weight and height were assessed on the day of admission for auto-HSCT. BMI and body surface area (BSA) were calculated using the calculation described in the supporting information (S1 Methods). According to the criteria of the world health organisation (WHO), normal weight was defined as a BMI<25, pre-obesity as a BMI between 25 and 30 and obesity if BMI was ≥30[20]; underweight (BMI≤20) was not separately analysed due to the small sample size of six patients in our cohort. During the study period all chemotherapy doses were capped at 2 m^2^ if calculated body surface area exceeded 2 m^2^. In consonance with the European Society for Clinical Nutrition and Metabolism, the Nutritional Risk Screening (NRS 2002) was assessed on the day of admittance. Patients with a NRS score ≥3 points were considered to be at high risk for malnutrition[21].

### Endpoints of the study

The predefined primary endpoint of the study was overall survival (OS). Secondary endpoints were progression free survival (PFS), NRM (defined as death not due to relapse or progression) and relapse incidence. The Frankfurt ethics committee approved the study (Approval number: SHN-5-2019) and informed written consent for the use of medical records was obtained. Standards of good clinical practice were followed during patient care and study conduct at all times.

### Statistical analysis

For statistical analysis SSPS version 25.0 (IBM Corp., SPSS Institute Inc. Chicago, USA) was used. Continuous variables were compared with the Mann-Whitney-U test, categorical variables the Fisher’s exact test and the chi-square test. Kaplan-Meier curves were plotted and compared by the Log-rank test. Cox proportional hazards were calculated for multivariate analysis. For competing risk analysis R version 3.3.2 was used (package “cmprsk”) and curves were compared by Gray’s test.

## Results

### Patient characteristics

Patient characteristics are displayed in Table 1.

**Table 1:** Patient characteristics.

Characteristics	All patients (n = 119)	BMI<30 (n = 94)	BMI ≥ 30 (n = 25)	p-value
Year of auto-HSCT, median (range)	2014 (2012–2016)	2014 (2012–2016)	2014 (2012–2016)	0.798
Male sex, n (%)	75 (60.0)	58 (61.7)	17 (68.0)	0.645
Age at auto-HSCT, median (range)	53 (19–75)	55 (19–75)	50 (29–69)	0.160
High-grade lymphoma, n (%)	94 (79.0)	73 (77.7)	21 (84.0)	0.590
B-cell lymphoma, n (%)	102 (85.7)	81 (86.2)	21 (84.0)	0.754
CNS-manifestation, n (%)	26 (21.8)	18 (19.1)	8 (32.0)	0.181
Ann Arbor Stage, n (%)				0.467
- 1	24 (20.2)	17 (18.1)	7 (28.0)	
- 2	15 (12.6)	12 (12.8)	3 (12.0)	
- 3	22 (18.5)	16 (17.0)	6 (24.0)	
- 4	58 (48.7)	49 (52.1)	9 (36.0)	
Months from diagnosis to auto-HSCT, median, (range)	13.27 (3.53–269.9)	15.2 (3.53–269.9)	12.1 (4.2–167.3)	0.395
Remissions status at auto-HSCT, n (%)				0.046
- Progressive disease	5 (4.2)	2 (2.1)	3 (12.0)	
- Stable disease	6 (5.0)	4 (4.3)	2 (8.0)	
- Partial remission	71 (59.7)	61 (64.9)	10 (40.0)	
- Complete remission	37 (31.1)	27 (28.7)	10 (40.0)	
Number of prior therapy lines, n (%)				0.776
- 1	36 (30.3)	27 (28.7)	9 (36.0)	
- 2	54 (45.4)	44 (46.8)	10 (40.0)	
- 3	27 (22.7)	21 (22.3)	6 (24.0)	
- 4	2 (1.7)	2 (2.1)	0	
ECOG performance score <2, n (%)	114 (95.8)	90 (95.7)	24 (96)	1.000
Diabetes, n (%)	5 (4.2)	3 (3.2)	2 (8.0)	0.282
HIV, n (%)	7 (5.9)	7 (7.4)	0	0.343
Lung disease, n (%)	4 (3.4)	3 (3.2)	1 (4.0)	1.000
Liver disease, n (%)	3 (2.5)	3 (3.2)	0	1.000
Heart disease, n (%)	13 (10.9)	10 (10.6)	3 (12.0)	1.000
Renal dysfunction, n (%)	5 (4.2)	4 (4.3)	1 (4.0)	1.000
Weight, kg, median (range)	78 (44.2–139.4)	71.5 (44.2–110)	103 (79–139.4)	<0.001
Height, cm, median (range)	174 (146–198)	173,5 (146–198)	175 (160–193)	0.407
BMI, median (range)	25.3 (17.7–46.4)	24 (17.7–29.9)	32.8 (30.0–46.4)	<0.001
BSA, median (range)	1.94 (1.405–2.629)	1.86 (1.405–2.449)	2.18 (1.84–2.63)	<0.001

p-values indicate differences between obese (BMI **≥** 30 and non-obese patients (BMI<30). auto-HSCT, autologous hematopoietic stem cell transplantation; CNS, central nervous system; ECOG, Eastern Cooperative Oncology Group; HIV, human immunodeficiency virus; BMI, body mass index; BSA, body surface area.

Overall, 119 predominantly male patients (n = 75, 60.0%) were included into this retrospective study with a median age of 53 years (range 19–75). In our study population, 25 patients (21.0%) were obese with a BMI≥30. Moreover, patients with a BMI<30 consisted of one subgroup with normal weight (BMI<25; n = 57; 47.9% of all patients) and one subgroup of pre-obese patients with a BMI between 25 and 30 (n = 37; 31.1% of all patients). The median weight of our cohort was 78 kg (range 44.2–139.4) and patients had a median body height of 174 cm (range 146–198), a median BMI of 25.3 (range 17.7–46.4) and a median BSA of 1.94 m^2^ (range 1.41–2.63). The median BMI in the group of obese patients was 32.8 (range 30.0–46.4) compared to the group of non-obese patients with a median BMI of 24.0 (range 17.7–29.9; p<0.001). The obese group had a significantly higher median body weight (103 kg vs. 71.5 kg; p<0.001) with no significant changes in the median body height (obese group: 175 cm vs. non-obese group: 173.5 cm; p = 0.407). In line with the higher body weight and BMI, the BSA of the obese patients was significantly greater (2.18 m^2^) compared to non-obese patients (1.86 m^2^; p<0.001)

A detailed description of the lymphoma subtypes in our study population is displayed in S1 Table and the distribution of obese and non-obese patients of the four main lymphoma-subtypes in our study is given in S2 Table. Most of the patients in our study (79.0%) suffered from high-grade lymphomas. The main subtype was B-cell lymphoma (85.7%). 26 patients (21.8%) had central nervous manifestations of their lymphomas. Most patients had an advanced Ann-Arbor stage with 48.7% suffering from stage IV disease. The median time from initial diagnosis to auto-HSCT was 13.3 months (range 3.5–269.9). Thirty-six patients (30.3%) received one prior therapy line (patients with an induction chemotherapy prior to upfront auto-HSCT), 45.4% two, 22.7% three and 1.7% four prior therapy lines before transplantation. A majority of patients (59.7%) achieved a partial remission, 31.1% a complete remission and 5.0% a stable disease during induction therapy prior to high-dose chemotherapy; five patients (4.2%) were transplanted while suffering from progressive disease. A large majority of patients (95.8%) received high-dose treatment at a performance status <2 (Eastern Cooperative Oncology Group, ECOG). The pre-existing medical conditions in the cohort included diabetes (4.2% of all patients), HIV-infections (5.9% of all patients), lung disease (3.4% of all patients), liver disease (2.5% of all patients), heart disease (10.9% of all patients) and renal dysfunction (4.2% of all patients). An overview of the medical conditions is given in S3 Table.

No differences between obese (BMI≥30) and non-obese patients (BMI<30) were found except for the remission status of the lymphoma at auto-HSCT. Significantly more patients had a stable or progressive disease in the group of obese patients (progressive disease: 12.0% vs. 2.1% and stable disease: 8.0% vs. 4.3%; p = 0.046). Differences in median age (50 vs. 55 years; p = 0.160) and CNS manifestations (32.0% vs. 19.1%; p = 0.181) were observed in the obese patients group but did not reach statistical significance.

### Transplant-related characteristics and outcomes

Table 2 summarizes the transplant-related characteristics of the study population. The median length of hospital stay for transplantation was 21 days (range 7–61). After transplantation, neutrophils recovered (>500/μl) with a median of 11 days (range 8–24) and platelets reached >50/μl after a median time of 17 days (range 10–160) post transplantation. No case of graft failure was observed. Weight loss and reduced food intake was assessed using the NRS 2002 scoring system, whereas 17.6% of all patients scored at ≥3. By statistical trend, more non-obese patients had an NRS 2002 score ≥3 (21.3% vs. 4%; p = 0.072). The median number of infused CD34+ cells was 2.73×10^6^/kg body weight. Fifteen patients (12.6%) had to be admitted to the intensive care unit in the peritransplant period, and 16 patients (13.4%) suffered from at least one BSI. No significant differences in the transplant related characteristics between both groups were observed.

**Table 2:** Transplant-related characteristics.

Characteristics	All patients (n = 119)	BMI<30 (n = 94)	BMI ≥ 30 (n = 25)	p-value
Neutrophil engraftment > 500/μl, days, median (range)	11 (8–24)	11 (8–24)	10 (9–29)	0.595
Platelet engraftment > 50/μl, days, median (range)	17 (10–160)	17 (10–160)	16 (12–55)	0.509
Hospital length of stay days, median (range)	21 (7–61)	22 (7–47)	21 (15–61)	0.259
NRS 2002 Score **≥** 3, n (%)	21 (17.6)	20 (21.3)	1 (4.0)	0.072
NRS 2002 Score–nutritional status, n (%)				0.398
- 0	94 (79.0)	72 (76.6)	22 (88.0)	
- 1	22 (18.5)	19 (20.2)	3 (12.0)	
- 2	3 (2.5)	3 (3.2)	0	
CD34+ cell dose (× 10^6^/kg), median (range)	2.73 (0.9–9.6)	2.74 (0.9–9.6)	2.73 (1.13–4.6)	0.439
Intensive care unit stay, n (%)	15 (12.6)	11 (11.7)	4 (16.0)	0.516
Bloodstream infection, n (%)	16 (13.4)	14 (14.9)	2 (8.0)	0.518

p-values indicate differences between obese (BMI ≥ 30 and non-obese patients (BMI<30). NRS, nutritional risk score.

An overview of patient outcomes is given in Table 3.

**Table 3:** Outcomes.

Characteristics	All patients (n = 119)	BMI<30 (n = 94)	BMI ≥ 30 (n = 25)	p-value
Estimated OS, % (95% CI)				0.005
- 3 months	93.9 (89.6, 98.2)	93.3 (88.2, 98.4)	96.0 (88.4, 100)	
- 12 months	83.1 (76.2, 90.0)	84.2 (76.6, 91.8)	78.9 (62.4, 95.4)	
- 48 months	71.3 (62.7, 79.9)	77.9 (69.1, 86.7)	45.3 (23.5, 67.1)	
Estimated PFS, % (95% CI)				<0.001
- 3 months	88.5 (82.6, 94.4)	89.9 (83.6, 96.2)	83.5 (68.6, 98.4)	
- 12 months	69.7 (61.3, 78.1)	73.9 (64.7, 83.1)	54.3 (34.3, 74.3)	
- 48 months	59.3 (49.9, 68.7)	67.2 (57.2, 77.2)	29.8 (10.0, 49.6)	
Cumulative incidence of relapse/progression	33.0 (23.4, 42.5)	21.5 (12.5, 30.5)	66.2 (44.6, 87.8)	<0.001
Cumulative incidence of NRM	9.7 (4.2, 15.2)	11.3 (4.6, 17.9)	4.0 (0, 11.8)	0.314

p-values indicate differences between obese (BMI **≥** 30 and non-obese patients (BMI<30). OS, overall survival; CI, confidence interval; PFS, progression free survival; NRM, non-relapse related mortality

**Fig 1:**
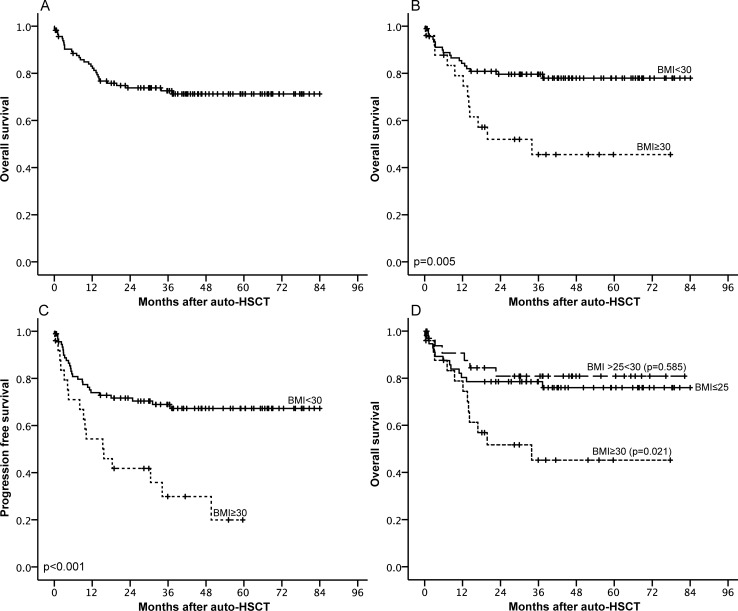
(A) Kaplan-Meier estimates for Overall survival (OS). (B) OS stratified by body mass index (BMI) <30 (solid line) and **≥**30 (dotted line). (C) Progression-free survival estimates stratified by BMI < 30 (solid line) and **≥** 30 (dotted line). (D) OS stratified by BMI ≤25 (solid line), >25 and <30 (dashed line) and **≥**30 (dotted line), p-values denote differences of each group compared to BMI ≤25.

The estimated overall survival of all patients (Fig 1A) was 71.3% (95% CI 62.7, 79.9). Obese patients displayed significantly lower OS levels compared to non-obese patients (Fig 1B): OS was 93.3% (95% CI 88.2, 98.4) at 3 months, and 77.9% (95% CI 69.1, 86.7) at 48 months in patients with a BMI<30. In obese patients (BMI≥30), OS was 96.0% (95% CI 88.4, 100) at 3 months, and 45.3% (95% CI 23.5, 67.1) at 48 months (p = 0.005).

The higher mortality in the obese group was mainly attributable to a significantly lower PFS rate (Fig 1C).

**Fig 2:**
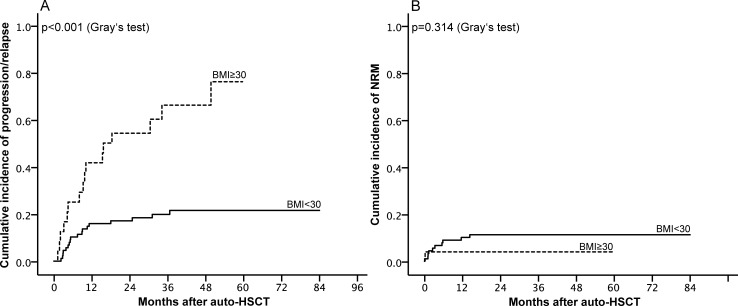
(A) Cumulative incidence of non-relapse related mortality (NRM) and (B) Cumulative incidence of relapse/progression, each stratified by BMI < 30 (solid line) and **≥** 30 (dotted line).

The PFS in the non-obese group declined from 89.9% (95% CI 83.6, 96.2) at 3 months to 67.2% (95% CI 57.2, 77.2) at 48 months, whereas we observed a way stronger decline in the group of obese patients (3 months: 83.5% [95% CI 68.6, 98.4] to 48 months: 29.8% [95% CI 10.0, 49.6]; p<0.001). In line with the differences in the OS and PFS, cumulative incidence of NRM (Fig 2A) did not differ significantly (p = 0.314) between obese and non-obese patients (non-obese: 11.3% vs. obese: 4.0%), but the cumulative incidence of relapse or progression (Fig 2B) was significantly higher in obese patients (non-obese: 21.5% vs. obese: 66.2%, p<0.001).

In a subgroup analysis of patients with DLBCL (representing the main lymphoma subtype in our study, n = 39) similar effects compared to the entire patient cohort were observed. The OS of non-obese DLBCL patients was 76.7% (95% CI 61.6, 91.8), whereas obese DLBCL patients displayed a significantly lower OS (28.6% [95% CI 0, 62.1], p = 0.036. The lower OS in this subgroup was also mainly attributable to a lower PFS rate (non-obese patients: 73.3% (95% CI 57.4, 89.2) vs. obese patients: 0% p = 0.007; S1 Fig). Comparing obese patients (BMI≥30) with normal weight (BMI<25) and pre-obese patients (BMI between 25–30; Fig 1D), pre-obese patients had a comparable OS to normal weight patients (pre-obese: 80.9% [95% CI 67.2, 94.6] and normal weight: 76.1% [95% CI 64.5, 87.7]; p = 0.585). However, obese patients revealed a significantly lower OS (45.3% 45.3 [95% CI 23.5, 67.1] compared to normal weight (p = 0.021) and pre-obese (p = 0.010) patients.

In a multivariate analysis for risk factors of death of any cause (Table 4), obesity was identified as an independent risk factor for death (HR 2.618 [95% CI 1.226–5.590]); p = 0.013) in the presence of age>65 years (HR 2.004 [95% CI 0.830–4.838]; p = 0.122), T-cell lymphoma subtype (HR 2.333 [95% CI 0.939–5.795]; p = 0.068), progressive/stable disease at auto-HSCT (HR 2.160 [95% CI 0.786–5.937]; p = 0.135) and a NRS 2002 score ≥3 (HR 1.234 [95% CI 0.496–4.303]; p = 0.492).

**Table 4:** Multivariate analysis of different risk factors for death of any cause.

	Multivariate analysis		
Characteristics	Hazard ratio	95% CI	P- value
Age > 65 years	2.004	0.830–4.838	0.122
T-cell lymphoma	2.333	0.939–5.795	0.068
BMI ≥ 30	2.618	1.226–5.590	0.013
NRS 2002 score ≥3	1.461	0.496–4.303	0.492
PD/SD at auto-HSCT	2.160	0.786–5.937	0.135

BMI, body mass index; ICU, intensive care unit; NRS, nutritional risk score; PD, progressive disease; SD, stable disease.

## Discussion

Obesity is associated with an inferior outcome in cancer patients[18, 22, 23]. Therefore, we evaluated the impact of obesity, defined as a BMI≥30, on the outcome of lymphoma patients receiving HDT followed by auto-HSCT in this retrospective study. Obesity was associated with a significant lower OS compared to non-obese patients. Interestingly, survival rates only separated after the peritransplant period (beyond three months after transplantation), and were accompanied by a separation of the PFS rates that showed a higher relapse rate among obese patients. In contrast, we did not observe differences in NRM between the two groups. Obese patients also displayed significantly lower OS when compared to normal weight (BMI<25) and pre-obese (BMI 25–30) patients. However, there were no significant differences in OS, when normal weight patients were compared with pre-obese patients. In a multivariate analysis, obesity was identified to be an independent risk factor for death in lymphoma patients undergoing auto-HSCT. Our study comprises a very homogenous patient cohort consisting only of lymphoma patients undergoing HDT and auto-HSCT (S2 Table) with well-balanced obese and non-obese patients in the different lymphoma subtypes (S2 Table). Most patients displayed advanced stage disease and received one or two prior lines of therapy. Baseline patient characteristics displayed no significant differences except for more patients with a progressive disease or stable disease at auto-HSCT in the group of obese patients. This might affect further interpretations of the different groups, especially in light of the fact that remission status at transplantation has been shown to be one of the most relevant indicators of long-term disease control[24, 25]. However, in the multivariate analysis obesity was identified with a clinically relevant hazard ratio (HR 2.618) as an independent risk factor for death, although disease remission status was included into the analysis.

In Western Europe, the prevalence of obesity has increased drastically and is currently approximated around 20%[26, 27]. Compared to previous studies (Tarella et al.: 77% BMI<28 and 23% BMI≥28 and Navarro et al.: 40% BMI<25, 36% BMI 25–30, 20% and BMI≥30), we report a similar distribution of normal weight (47%), pre-obesity (32%) and obesity (21%) indicated by BMI in our patient population compared to both studies in the setting of stem cell transplantation[17, 18] as well as compared to the general prevalence of 20%. Pre-obesity and obesity are associated with increased comorbidity rates and a poorer overall health status compared to adults with normal body weight[28]. An altered health status may result in impaired outcome in the obese patient group compared to the normal weight group. However, the ECOG performance status did not differ significantly in our study population. Furthermore, obesity in general is associated with diabetes as well as cardiovascular diseases[28, 29]. Again, no higher rates of diabetes and heart disease in the obese group of our study population could be observed. In general, the frequency of diabetes in our cohort (4.2%) was lower compared to the reported European incidence of 10%[30]. Since only medically fit patients were considered for HDT and auto-HSCT, this may explain the lower incidence of diabetic comorbidity in our study compared to the reported European incidence.

Furthermore, not all risk factors and pre-existing conditions such as pre-diabetic state or disturbances of lipid metabolism that may indicate metabolic syndrome can be assessed in a retrospective study. Therefore, potential causes of obesity related mortality may remain unclear and no conclusions regarding their impact on clinical outcome in lymphoma patients undergoing auto-HSCT may be drawn.

Regarding the treatment related toxicities, the incidence of BSIs, ICU admissions and duration of hospital stay did not differ between the obese and non-obese patients in our study. This is in line with results from previous studies, where infection rates as well as median inpatient stay time did not differ between normal weight and obese lymphoma patients during HDT and auto-HSCT[17, 18].

In the present analysis, we show that obesity was associated with an impaired overall survival and increased rates of relapse in lymphoma patients undergoing HDT followed by auto-HSCT. Multiple factors contribute to this finding. Obesity has been shown to result in chronic inflammation and further increased onset and progression of cancer[31–33]. In obesity-associated cancer types such as endometrial carcinoma or breast cancer, obesity is believed to favour tumour progression via the adipose tissue microenvironment and further altered endocrine state[31]. By endocrine and paracrine affection, adipose tissue might directly enhance tumour growth and angiogenesis. Additionally, the antiapoptotic effect of obesity-related hyperinsulinemia is thought to further promote tumour progression[34, 35]. This may also contribute to impaired overall survival due to a higher relapse incidence in the obese patient group in our study.

Furthermore, one factor that might impact the clinical outcome after HDT and auto-HSCT is the dosage of chemotherapeutic agents used in HDT regimens and during previous induction chemotherapy. Underdosage of chemotherapy in obese patients may contribute to increased mortality after treatment in obese patients[36]. Currently, the BSA is widely used to determine the individual dosage for cancer patients undergoing chemotherapy[37]. Other calculation models use the actual body weight, the idealised bodyweight (IBW) or the adjusted IBW (AIBW)[38]. To limit chemotherapeutic toxicities in obese patients, chemotherapy dosage is often reduced by not exceeding a set maximum of BSA for calculation[36, 38, 39]. Patients in our study cohort received HDT calculated by BSA. The maximum BSA used for dosage calculation was 2 m^2^ to prevent obese patients from increased therapy-related toxicity. In this regard, it has been reported that severe oral mucositis–one major clinical toxicity of HDT and known risk factor for infections–was increased with higher dose/weight ratio in NHL patients receiving carmustine[40]. Other toxicities that are exemplarily known to increase with dose are etoposide induced mucositis or carmustine associated pulmonary toxicity[41]. Moreover, obese patients seem to be at a higher risk for infections and hospitalisation rates during and after allogeneic stem cell transplantation[42] and a higher NRM after auto-HSCT with similar relapse rates was reported[43]. Using dose limiting to prevent increased toxicity without loss in antitumor effect seems feasible due to previous results on the association of dose/weight relation with adverse side effects in DLBCL patients undergoing conditioning chemotherapy for auto-HSCT[44]. Additionally, in a large recently published study in lymphoma and multiple myeloma patients undergoing auto-HSCT, no increased relapse rates were observed concerning dose reductions[45].

Contrarily, in most solid tumours, the guidelines recommend calculating the BSA based on the actual body weight[46] and evident data is published, that obese patients may be repeatedly undertreated and do not benefit from dose reduction or chemotherapy capping[14, 36, 46, 47]. Furthermore, obese NHL patients tolerate uncapped chemotherapy dosing and showed similar treatment-related toxicities compared to non-obese NHL patients[48]. Level I or II evidence-based dosing recommendations are missing for HDT followed by auto-HSCT. Currently, our clinical center does not further cap the BSA at 2 m^2^ according to the consensus recommendations of the American Society for Blood and Marrow Transplantation. Instead, we now calculate chemotherapy dosage either by uncapped BSA, absolute body weight, IBW or AIBW depending on the chemotherapeutic agent[41] since we identified obese lymphoma patients to have an impaired overall survival. However, large prospective clinical trials are required, to further address the impact of obesity on the clinical outcome of HDT followed by auto-HSCT.

The limitations of our retrospective study prevent from drawing causal conclusions from its results. Moreover, all 119 patients in our study all had lymphoma and were treated with HDT and auto-HSCT. Thus, general conclusions for PFS and OS post auto-HSCT in other malignancies cannot be made. A detailed study on obesity-related comorbidities, pharmacodynamics of chemotherapeutic agents in obese patients, sarcopenia and quantity of adipose tissue as well as an evaluation of prevalence and severity of toxicities with and without chemotherapy dose-capping in the context of auto-HSCT may provide further insight into the poor clinical outcomes of obese patients in comparison with non-obese patients.

In conclusion, the present study reveals that obesity is associated with impaired OS due to a higher incidence of relapse in lymphoma patients treated with HDT and concomitant auto-HSCT. Further studies will be required to fully elucidate this negative impact of obesity on patient outcome.

## Supporting information

S1 Fig Subgroup analysis on overall survival (A) and progression free survival (B) of patients with DLBCL.
(EPS)

S1 Methods Calculation formulas of body mass index and body surface area. (DOCX)

S1 Table Lymphoma subtypes. (DOCX)

S2 Table Distribution of obese and non-obese patients of four main lymphoma subtypes. (DOCX)

S3 Table Pre-existing medical conditions. (DOCX)
