# Supplementary material for: Obesity is associated with an impaired survival in lymphoma patients undergoing autologous stem cell transplantation
Source: PLoS One. 2019 Nov 8;14(11):e0225035. doi: 10.1371/journal.pone.0225035 (PMC6839865; doi:10.1371/journal.pone.0225035)
Supplement: S1 Methods — (DOCX) [file pone.0225035.s002.docx]

**S1 Methods.**

Calculation of body mass index

$$BMI=\frac{{mass}_{kg}}{{height}_{m}^{2}}$$

Calculation of body surface area (BSA, DuBois formula)

$$BSA=0.007184m^{2}\times\left( \frac{height}{cm} \right)^{0.725}\times\left( \frac{mass}{kg} \right)^{0.425}$$
