# Supplementary material for: Obesity is associated with an impaired survival in lymphoma patients undergoing autologous stem cell transplantation
Source: PLoS One. 2019 Nov 8;14(11):e0225035. doi: 10.1371/journal.pone.0225035 (PMC6839865; doi:10.1371/journal.pone.0225035)
Supplement: S1 Table — (DOCX) [file pone.0225035.s003.docx]

**S1 Table.**

| **Lymphoma subtype** | **Number (% of all patients)** |
| --- | --- |
| Diffuse large B cell lymphoma | 39 (32.8) |
| Primary CNS lymphoma | 21 (17.6) |
| Angioimmunoblastic T cell lymphoma | 1 (0.8) |
| Anaplastic T cell lymphoma | 7 (5.9) |
| Burkitt lymphoma/lymphoblastic lymphoma | 2 (1.7) |
| Follicular lymphoma | 9 (7.6) |
| Grey zone lymphoma | 2 (1.7) |
| Hodgkin lymphoma | 12 (10.1) |
| Lymphoplasmacytic lymphoma | 1 (0.8) |
| Mantle cell lymphoma | 14 (11.8) |
| NK T cell lymphoma | 3 (2.5) |
| Peripheral T cell lymphoma | 5 (4.2) |
| Primary mediastinal B cell lymphoma | 1 (0.8) |
| T cell rich B cell lymphoma | 2 (1.7) |
