# Supplementary material for: Obesity is associated with an impaired survival in lymphoma patients undergoing autologous stem cell transplantation
Source: PLoS One. 2019 Nov 8;14(11):e0225035. doi: 10.1371/journal.pone.0225035 (PMC6839865; doi:10.1371/journal.pone.0225035)
Supplement: S2 Table — (DOCX) [file pone.0225035.s004.docx]

**S2 Table.**

| Lymphoma subtype | All patients  (n = 119) | BMI<30 (n=94) | BMI **≥** 30  (n=25) | p-value |
| --- | --- | --- | --- | --- |
| Diffuse large B cell lymphoma | 39 (32.8) | 32 (34.0) | 7 (28.0) | 0.638 |
| Primary CNS lymphoma | 21 (17.6) | 14 (14.9) | 7 (28.0) | 0.145 |
| Mantle cell lymphoma | 14 (11.8) | 12 (12.8) | 2 (8.0) | 0.731 |
| Hodgkin lymphoma | 12 (10.1) | 9 (9.6) | 3 (12.0) | 0.714 |
