# Supplementary material for: Obesity is associated with an impaired survival in lymphoma patients undergoing autologous stem cell transplantation
Source: PLoS One. 2019 Nov 8;14(11):e0225035. doi: 10.1371/journal.pone.0225035 (PMC6839865; doi:10.1371/journal.pone.0225035)
Supplement: S3 Table — (DOCX) [file pone.0225035.s005.docx]

**S3 Table.**

|  | **Description (number)** |
| --- | --- |
| Heart disease | Coronary heart disease (4)  Atrial fibrillation (4)  Congestive heart failure (4)  Sinuatrial block (1) |
| Lung disease | Chronical obstructive pulmonary disease (2), Sarcoidosis (1)  Pulmonary embolism (1) |
| Liver disease | Hepatitis B (2)  Hepatitis C (1) |
